# Impairment of written word production in logopenic primary progressive aphasia: dissociating phonological and orthographic contributions

**DOI:** 10.3389/fnhum.2026.1812997

**Published:** 2026-04-07

**Authors:** Joël Macoir, Robert Laforce, Monica Lavoie

**Affiliations:** 1 Faculté de Médecine, École des Sciences de la Réadaptation, Université Laval, Québec, QC, Canada; 2 Centre de Recherche CERVO—Brain Research Centre, Québec, QC, Canada; 3 Chaire de recherche sur les aphasies primaires progressives – Fondation de la Famille Lemaire, Québec, QC, Canada; 4 Clinique Interdisciplinaire de Mémoire (CIME) du CHU de Québec, Département des Sciences Neurologiques, Québec, QC, Canada

**Keywords:** agraphia, neurodegenerative language disorders, orthographic processing, primary progressive aphasia (logopenic variant), written language impairment

## Abstract

**Introduction:**

Written language impairment is a frequent but still incompletely understood feature of the logopenic variant of primary progressive aphasia (lvPPA), and although spoken language deficits have been extensively studied, less is known about the mechanisms underlying impaired written word production and the conditions under which orthographic representations become particularly vulnerable. In this study, we investigated written language processing in individuals with lvPPA using a battery of tasks designed to dissociate phonological and orthographic contributions.

**Methods:**

Twelve participants with lvPPA and thirteen healthy controls completed written picture naming, written word dictation, written non-word dictation, grapheme dictation, and a picture-based letter-in-word judgment task that probed orthographic knowledge without requiring overt written output.

**Results:**

Compared with controls, participants with lvPPA showed significant impairments in written picture naming, written word dictation, grapheme dictation, and letter-in-word judgment, whereas written non-word dictation was relatively preserved at the group level. Within grapheme dictation, vowel graphemes were disproportionately affected relative to consonants. In the letter-in-word judgment task, the largest group differences were observed in conditions requiring access to orthographic knowledge when phonological cues were non-informative.

**Discussion:**

Together, these findings indicate that written language impairment in lvPPA cannot be explained by a primary phonological deficit alone but instead reflects vulnerability of abstract orthographic representations, particularly under conditions of increased orthographic ambiguity. Identifying tasks that require access to orthographic knowledge independently of phonological support may therefore provide sensitive markers for characterizing written language impairment in lvPPA and contribute to a more precise understanding of language network dysfunction in neurodegenerative disease.

## 1 Introduction

Primary progressive aphasia is a neurodegenerative syndrome characterized by a gradual and selective decline of language functions, with relative preservation of other cognitive domains in the early stages of the disease (Mesulam, 2003, 2013). Current consensus criteria distinguish three main clinical variants: the non-fluent/agrammatic variant, the semantic variant, and the logopenic variant (Gorno-Tempini et al., 2011). Among these, the logopenic variant of primary progressive aphasia (lvPPA) is typically associated with impaired word retrieval, reduced word and sentence repetition, and deficits in phonological short-term memory, reflecting predominant involvement of left temporoparietal language networks (Gorno-Tempini et al., 2008; Henry and Gorno-Tempini, 2010).

While spoken language impairments in lvPPA have been extensively documented, written language production has received comparatively less systematic attention. This is notable given that writing is frequently affected early in the disease course and may provide sensitive markers of underlying language system disruption (Graham, 2014; Neophytou et al., 2019). Moreover, written language offers a unique window onto the organization of lexical, phonological, and orthographic representations, allowing dissociation of processing components that are more difficult to isolate in spoken production.

From a neuropsychological perspective, written word production is supported by multiple interacting mechanisms, including lexical–semantic access, phonological processing, phoneme–grapheme conversion, access to abstract orthographic representations, and the temporary maintenance of graphemic information during serial output (Tainturier and Rapp, 2001; Rapp and Fischer-Baum, 2014; Rapp and Purcell, 2019). Disruption at different levels of this system gives rise to distinct patterns of acquired agraphia, traditionally classified as phonological, surface, or deep agraphia (Denes et al., 2020). Although these classifications were originally derived from focal lesion studies, they continue to provide a useful conceptual framework for characterizing writing impairments in neurodegenerative conditions, including PPA (Graham, 2000, 2014).

Existing studies of spelling and writing in lvPPA have reported heterogeneous profiles that do not map neatly onto a single agraphic syndrome. Group studies and case series have documented impairments affecting both word and non-word spelling, often accompanied by a mixture of phonologically plausible errors (e.g., bato for bateau, “boat”) and non-phonologically plausible errors (e.g., bameau for bateau) (Sepelyak et al., 2011; Shim et al., 2012; Faria et al., 2013). Such patterns suggest that spelling deficits in lvPPA may reflect disruptions at multiple levels of the spelling system rather than a selective breakdown of a single route.

Importantly, these written language impairments occur within the broader language profile of lvPPA, which is characterized by impaired phonological short-term memory, reduced repetition, and word-finding difficulties associated with temporoparietal network degeneration (Gorno-Tempini et al., 2008; Henry and Gorno-Tempini, 2010). Because written word production requires coordinated access to phonological, lexical, and orthographic representations, deficits affecting phonological processing or lexical retrieval may propagate to written production, while additional impairments in orthographic representations may further compromise spelling accuracy.

Sensitivity to psycholinguistic variables such as lexical frequency, orthographic regularity, word length, and semantic properties has also been observed, highlighting the contribution of lexical–orthographic knowledge to written production deficits in lvPPA (Neophytou et al., 2019; Carroll-Duhigg et al., 2025). Together, these findings suggest that agraphia in lvPPA reflects disruption of multiple interacting components of the writing system rather than a uniform breakdown of a single spelling route.

However, much of the existing literature has relied on global accuracy measures or broad lexicality contrasts (e.g., words vs. non-words), which may obscure more selective vulnerabilities within orthographic processing. In particular, the availability and stability of abstract orthographic representations in lvPPA remain poorly understood, especially under conditions in which phonological information provides limited or unreliable support. According to influential models of spelling, accurate written production relies on access to abstract orthographic representations that cannot be derived directly from phonology, particularly in languages characterized by inconsistent phoneme–grapheme mappings (Rapp and Caramazza, 1997). Although sublexical phoneme–grapheme conversion processes are sensitive to the statistical regularities of the spelling system (Ziegler et al., 1996; Peereman and Content, 1999), they are insufficient to support accurate spelling in many cases, especially when mappings are of low consistency, thereby necessitating the contribution of lexical–orthographic knowledge (Barry and Seymour, 1988).

This issue is especially relevant in French, where phoneme to grapheme correspondences is highly inconsistent in the phonology-to-orthography direction (Ziegler et al., 1996), and where vowel phonemes are associated with substantial orthographic ambiguity (Jaffré and Fayol, 2013). For example, the French vowel phoneme /o/ can correspond to several orthographic forms (e.g., o, au, eau), and the phoneme /ε/ may be spelled in, ain, or ein. Such variability increases the demands placed on orthographic selection processes because phonological information alone does not uniquely determine the correct spelling. As a result, vowel spelling places disproportionate demands on orthographic selection processes and on the integrity of abstract orthographic representations, beyond what can be achieved through sublexical phonological conversion alone. Consistent with this view, neuropsychological evidence indicates that consonants and vowels are represented as distinct categories within the orthographic system and can be selectively impaired following brain damage (Buchwald and Rapp, 2006). Vulnerability at this level may therefore be particularly informative for identifying orthographic impairments in lvPPA.

Tasks that explicitly manipulate the relationship between phonological and orthographic information offer a powerful means of probing these mechanisms. Letter-based judgment tasks, in which participants decide whether a given letter belongs to the written form of a word, have been shown to tap orthographic knowledge independently of overt written output (Macoir and Beland, 2004; Purcell and Rapp, 2013). By crossing the presence or absence of a target letter in the orthographic form with the presence or absence of the corresponding phoneme in the phonological form, such tasks allow fine-grained examination of conditions in which orthographic decisions can or cannot be supported by phonological cues. Despite their theoretical relevance, these paradigms have rarely been applied to the study of written language impairment in lvPPA. In particular, identifying conditions under which orthographic representations must be accessed independently of phonological support may provide especially sensitive markers of written language impairment in lvPPA.

The present study aimed to provide a detailed characterization of written word production deficits in lvPPA by combining global task-level analyses with targeted manipulations designed to probe orthographic processing and phonology–orthography interactions. Using a battery of written production tasks, including written picture naming, written word dictation, written non-word dictation, grapheme dictation, and a picture-based letter-in-word judgment task, we examined both overall accuracy and qualitative error patterns in individuals with lvPPA compared to healthy control participants. Particular emphasis was placed on dissociating lexical and sublexical spelling mechanisms, assessing the influence of psycholinguistic variables known to tax orthographic representations, and identifying conditions in which access to orthographic knowledge was required independently of phonological support.

Based on current cognitive models of spelling (Rapp and Caramazza, 1997; Tainturier and Rapp, 2001) and previous observations in lvPPA, two main hypotheses were formulated. First, we predicted that individuals with lvPPA would show disproportionate difficulty in tasks requiring access to orthographic knowledge when phonological information provides limited support. Second, given the high degree of phoneme–grapheme ambiguity associated with French vowel phonemes, we expected vowel graphemes to be particularly vulnerable relative to consonant graphemes. These predictions guided the selection of tasks designed to dissociate phonological and orthographic contributions to written word production.

By adopting this fine-grained approach, the present study also sought to clarify the nature of agraphia in lvPPA and to determine whether specific components of the orthographic system, most notably those involved in vowel processing and phonology-independent orthographic access, constitute points of particular vulnerability in this variant. Beyond contributing to cognitive models of written language production, this work also aimed to inform clinical assessment by identifying tasks and conditions that were especially sensitive to written language impairment in lvPPA.

## 2 Method

### 2.1 Participants

Thirteen individuals meeting diagnostic criteria for the logopenic variant of primary progressive aphasia (lvPPA) were initially recruited through the Clinique Interdisciplinaire de Mémoire (CIME) at the Centre Hospitalier Universitaire (CHU) de Québec. All participants were evaluated and diagnosed by a neurologist specializing in neurodegenerative cognitive disorders, in accordance with the consensus criteria proposed by Gorno-Tempini et al. (2011). Diagnosis was based on a comprehensive clinical language assessment and neurological examination and was supported by available biomarkers in 12 of the 13 patients.

Diagnostic support was obtained from structural and/or functional neuroimaging findings and, when available, biological biomarkers derived from cerebrospinal fluid or plasma. Specifically, four patients underwent FDG-PET (fluorodeoxyglucose positron emission tomography); two underwent FDG-PET combined with lumbar puncture; one underwent FDG-PET combined with PrecivityAD2 testing; three underwent computed tomography (CT); one underwent CT combined with lumbar puncture; and one underwent magnetic resonance imaging (MRI). When available, neuroimaging findings were consistent with the typical pattern associated with lvPPA, including predominant involvement of left temporoparietal language regions. In FDG-PET examinations, this corresponded to reduced metabolic activity in left temporoparietal areas, whereas structural imaging (MRI or CT) revealed atrophy patterns compatible with degeneration of the same network. These imaging findings were interpreted by the treating neurologist as supportive of the clinical diagnosis in accordance with the consensus criteria for primary progressive aphasia (Gorno-Tempini et al., 2011).

All participants exhibited a clinical profile characteristic of lvPPA, including prominent word-finding difficulties, impaired word and sentence repetition, and reduced phonological short-term memory, in the absence of motor speech disorders or predominant semantic impairment. A comprehensive medical and psychiatric history was obtained for each participant. Individuals were excluded if they had a history of neurological or cerebrovascular disease other than lvPPA, any current or past psychiatric illness as defined by the DSM-5 (American Psychiatric Association, 2013), traumatic brain injury, untreated medical or metabolic conditions (e.g., diabetes or thyroid dysfunction), prior intracranial surgery, or uncorrected auditory or visual impairments.

The comparison group consisted of 13 neurologically healthy adults recruited through the Centre de recherche CERVO. Control participants were excluded if they met any of the following criteria: (1) performance below a Z score of −1.33 on the Montreal Cognitive Assessment (MoCA; Nasreddine et al., 2005), based on normative standards established by the research team (Larouche et al., 2016); (2) performance below the fifth percentile (approximately 1.5 standard deviations below the mean) on the Detection Test for Language Impairments in Adults and the Aged (DTLA; Macoir et al., 2017, 2022); (3) presence of an active or unstable psychiatric condition; (4) history of moderate to severe traumatic brain injury; (5) prior stroke or other neurological disorder; (6) history of psychotic illness; (7) substance abuse; or (8) uncorrected auditory or visual impairment. These exclusion criteria were applied to reduce the risk of including individuals with subtle or preclinical cognitive deficits that could confound group comparisons. As reported in Table 1, the control and lvPPA groups were comparable in age, sex distribution, and years of formal education. Time since diagnosis for participants with lvPPA is also reported in Table 1. At the time of testing, patients had received their diagnosis an average of 8.75 months earlier (range = 2–23 months), indicating that most participants were assessed relatively early in the course of the disease.

**Table 1:** Sample characteristics and background cognitive measures in lvPPA and healthy control groups.

Variables	HC (*n* = 13)		lvPPA (*n* = 12)				
	*M* (SD)	min-max	*M* (SD)	min-max	*U/χ^2^*	*p*	*r*
Demographics							
Age	69.85 (6.67)	59–82	70.08 (7.88)	55–82	*U =* 79.5	0.817	0.050
Sex (male/female)	5/8	–	5/7	–	*χ^2^*(1) = 0.03	0.87	–
Education (years)	14.85 (2.34)	11–18	14.69 (2.36)	11–18	*U =* 88.5	0.854	0.040
Time from diagnosis (months)	–	–	8.75 (6.68)	2–23	–	–	–
Cognitive screening							
MoCA	27.46 (1.76)	25–30	15.85 (7.57)	5–30	*U =* 151.5	0.001	0.674
DTLA	95.23 (3.56)	90–100	69.00 (17.64)	40–96	*U =* 162.0	< 0.001	0.779
Verbal short-term and working memory							
Short-term memory (digit span forward)	5.92 (0.64)	5–7	4.31 (1.25)	3–6	*U =* 143.5	0.002	0.593
Working memory (digit span backward)	4.08 (0.64)	3–5	2.23 (1.01)	0–4	*U =* 159.0	< 0.001	0.749
Executive functions							
Alphaflex time A (sec.)	8.54 (2.60)	6–15	21.85 (27.47)	7–110	*U =* 32.0	0.007	0.528
Alphaflex error A	0.46 (0.66)	0–2	4.23 (6.29)	0–18	*U =* 63.5	0.241	0.211
Alphaflex time B (sec.)	17.77 (4.11)	12–27	34.62 (22.70)	20–90	*U =* 14.0	< 0.001	0.709
Alphaflex error B	0.69 (0.755)	0–2	4.77 (4.71)	0–12	*U =* 39.0	0.017	0.458

Values represent means (standard deviations) and ranges. Group comparisons were performed using Mann–Whitney U-tests due to non-normal distributions in the lvPPA group, while comparison for sex was made using the χ^2^ test. Effect sizes are reported as r, calculated from the standardized U statistic, with values of approximately 0.10, 0.30, and 0.50 interpreted as small, medium, and large effects, respectively. Higher scores indicate better performance except for Alphaflex completion times and error counts, for which lower values reflect better performance.

During the clinical interview, participants were also asked about their medical and educational history. None reported a history of developmental reading or writing disorders (e.g., dyslexia or dysgraphia), and the comparable levels of formal education observed in the lvPPA and control groups (Table 1) further suggest typical premorbid literacy acquisition.

One participant in the lvPPA group exhibited extremely low performance across written word production tasks. This individual was a 64-year-old man with 18 years of education who had received a diagnosis of lvPPA 15 months prior to testing. Although global cognitive screening remained preserved (MoCA = 26), language screening revealed severe impairment (DTLA = 40), accompanied by marked deficits in verbal short-term and working memory (digit span forward = 3; backward = 0) and executive functioning (Alphaflex A time = 110 s, errors = 15; Alphaflex B time = 20 s, errors = 12). Written language performance was near floor level across most tasks (written picture naming = 2/24; written word dictation = 1/47; written non-word dictation = 0/15; grapheme dictation = 8/30), although performance remained relatively preserved on the letter-in-word judgment task (19/24). Because this performance profile precluded meaningful interpretation of the targeted linguistic effects and exerted a disproportionate influence on group-level statistical analyses, this participant was excluded from inferential analyses.

Inferential statistical analyses were therefore conducted on a final sample of 12 individuals with lvPPA and 13 healthy control participants. All individuals gave written informed consent after receiving a full explanation of the study procedures. Ethical approval was obtained from the Ethics Committee for the Sector Research in Neurosciences and Mental Health (Project MP-13-2017-164).

### 2.2 Materials

#### 2.2.1 Cognitive screening

Overall cognitive status was examined using the MoCA (Nasreddine et al., 2005), a standardized instrument commonly employed for the detection of mild cognitive impairment. The MoCA provides a brief assessment of several cognitive domains, including attention, executive functioning, memory, language, visuospatial abilities, and temporal and spatial orientation. Administration followed standard guidelines, yielding a total score ranging from 0 to 30.

Language functioning was evaluated with the DTLA (Macoir et al., 2017, 2022). This short screening battery was specifically designed to identify language deficits associated with neurodegenerative conditions. It comprises nine tasks probing key components of language processing, such as confrontation naming, repetition of words and non-words, verbal fluency, reading and spelling, sentence comprehension, semantic processing, and an alphabetization span task indexing verbal working memory. Together, these DTLA measures provide a brief yet sensitive overview of both receptive and expressive language abilities across multiple linguistic levels.

In addition to global cognitive screening tools, tests of verbal short-term and working memory as well as executive functions were administered. Verbal short-term and working memory was assessed using the Digit Span subtest from the Wechsler Adult Intelligence Scale–Fourth Edition (WAIS-IV; Wechsler, 2008). Participants completed both the forward and backward conditions, administered according to standardized procedures. The forward condition required immediate repetition of auditorily presented digit sequences in their original order and served as an index of simple verbal span. The backward condition involved reproducing the digits in reverse order, thereby placing additional demands on mental manipulation and working memory. Performance was quantified as the maximum sequence length correctly recalled for each condition.

Executive functioning was examined with the Alphaflex (Grotz et al., 2018), a brief orally administered task designed to assess reactive mental flexibility with minimal visuomotor involvement and comprising two conditions. In Part A, participants recited the alphabet aloud as quickly and accurately as possible, providing a baseline measure of an overlearned verbal sequence. In Part B, they were instructed to produce only every second letter of the alphabet (e.g., A, C, E), a manipulation that increases demands on inhibitory control, sequencing, and internal monitoring. For each condition, completion time, and number of errors were recorded.

Together, these measures provided a comprehensive characterization of the participants' global cognitive, language, and executive profiles.

#### 2.2.2 Battery of written word production tasks

Written language production was assessed using a targeted battery designed specifically to examine key components of written word production in lvPPA. The selected tasks probed complementary stages of the spelling architecture, including lexical–semantic access, phonological-to-orthographic conversion, orthographic output processes, and serial grapheme selection. Together, these tasks allowed for a fine-grained characterization of both accuracy and error patterns in written production.

The battery comprised five tasks: written picture naming, written word dictation, written non-word dictation, grapheme dictation, and a letter-in-word judgment task based on picture stimuli. Although no written output is required, successful performance on the letter-in-word judgment task presupposes the internal generation and evaluation of the target word's orthographic representation, thereby engaging central spelling processes involved in written word production rather than mere visual or recognition-based mechanisms. A detailed overview of each task, including the number of items, controlled linguistic parameters, scoring procedures, and targeted cognitive processes, is provided in Table 2.

**Table 2:** Overview of the written word production tasks, controlled linguistic parameters, and targeted cognitive processes.

Task	Description	Number of items	Controlled parameters	Primary cognitive processes targeted	Scoring
Written picture naming	Writing the name of visually presented objects	24	Lexical frequency; orthographic regularity; syllabic length	Lexical–semantic access; orthographic output lexicon; graphemic buffer; serial grapheme production	1 point per correctly written word (max = 24); error analysis
Written word dictation	Writing auditorily presented real words	47	Lexical frequency; imageability; orthographic regularity; syllabic length	Lexical and sublexical spelling routes; phonological-to-orthographic conversion; orthographic working memory	1 point per correctly spelled word (max = 47); error analysis
Written non-word dictation	Writing auditorily presented non-words	15	Syllabic length	Sublexical phonological-to-orthographic conversion; graphemic buffer	1 point per acceptable spelling (max = 15); error analysis
Grapheme dictation	Writing graphemes corresponding to spoken phonemes	30	Grapheme type	Phoneme-to-grapheme conversion; sublexical spelling mechanisms	1 point per correct response (max = 30); error analysis
Letter-in-word judgment (picture-based)	Judging whether a given letter belongs to the written form of a pictured word	24 (4 × 6)	Lexical frequency; presence/absence of target letter and corresponding phoneme	Orthographic output lexicon access; orthographic knowledge without written output	1 point per correct judgment (max = 24)

All tasks were administered following standardized procedures. Accuracy scores reflect the number of correct responses. In addition to quantitative scores, written responses were transcribed verbatim to allow qualitative classification of error types. The letter-in-word judgment task was subdivided into four conditions based on the presence or absence of the target letter in the orthographic form and the corresponding phoneme in the phonological form (6 items per condition): (a) phoneme present/letter present, (b) phoneme absent/letter present, (c) phoneme present/letter absent, and (d) phoneme absent/letter absent.

Briefly, written picture naming assessed access to orthographic representations from visual–semantic input, while written word dictation examined spelling mechanisms under auditory input, with systematic manipulation of lexical frequency, orthographic regularity, syllabic length, and imageability. Written non-word dictation and grapheme dictation were included to specifically evaluate sublexical phonological-to-orthographic conversion processes independent of lexical knowledge. Finally, the letter-in-word judgment task provided a measure of orthographic knowledge without requiring full written output, thereby isolating access to the orthographic output lexicon.

All tasks were administered following standardized procedures. Accuracy scores were computed for each task, and all responses were transcribed verbatim to enable qualitative classification of error types, including phonologically plausible and non-phonologically plausible errors, substitutions, perseverations, and omissions.

### 2.3 Procedure

All participants were evaluated individually either in a quiet room in their home or in a dedicated testing space at the research center. Assessments were carried out by trained research assistants under the supervision of the primary investigator, and each evaluation was completed in a single session lasting approximately 90 min.

Testing followed a standardized sequence to minimize potential task interference. Participants first completed the MoCA and the DTLA, after which they were administered the digit span tasks followed by the Alphaflex. The session concluded with the administration of the battery of written word production tasks. All responses were scored immediately according to the standardized scoring criteria, and data were subsequently entered into a secure database for analysis. Recruitment and testing occurred between February 2023 and October 2025.

### 2.4 Statistical analyses

All statistical analyses were conducted using Jamovi (version 2.6.45; The Jamovi Project, 2025). Given the relatively small sample size and the presence of non-normal and bounded score distributions across several measures, non-parametric statistical methods were used throughout.

Between-group comparisons were first conducted on cognitive screening and diagnostic measures, including global cognitive status (MoCA), language screening (DTLA), verbal short-term and working memory (digit span forward and backward), and executive function measures (Alphaflex completion times and error counts for Parts A and B). Group differences were examined using Mann–Whitney *U*-tests due to non-normal score distributions. Effect sizes were reported as *r*. These analyses were intended to characterize group-level cognitive differences and were not adjusted for multiple comparisons.

#### 2.4.1 Quantitative analyses of overall task performance

Between-group differences in written word production were first examined using total accuracy scores for each task, including written picture naming, written word dictation, written non-word dictation, grapheme dictation, and the letter-in-word judgment task. Group comparisons were performed using Mann–Whitney *U-*tests. Effect sizes were reported as *r*, calculated from the standardized U statistic, with values of approximately 0.10, 0.30, and 0.50 interpreted as small, medium, and large effects, respectively. To control for multiple comparisons across tasks, *p-values* were adjusted using the Holm–Bonferroni procedure.

In a second step, the letter-in-word judgment task was further decomposed into four subcategories defined by the presence or absence of the target letter in the orthographic form and the corresponding phoneme in the phonological form. Between-group comparisons were conducted separately for each subcategory using Mann–Whitney *U*-tests. To control for multiple comparisons within this set of related analyses, *p-values* were adjusted using the Holm–Bonferroni procedure applied across the four subcategories.

Finally, to further characterize performance on grapheme dictation, accuracy scores were examined separately for consonant and vowel graphemes. Between-group comparisons were conducted using Mann–Whitney *U*-tests and were interpreted as secondary, targeted analyses.

#### 2.4.2 Influence of psycholinguistic parameters

To investigate the influence of psycholinguistic variables on written production performance, additional analyses were conducted for tasks in which these parameters were systematically manipulated. Within each group, the effects of lexical frequency, orthographic regularity, and syllabic length were examined using Wilcoxon signed-rank tests. To assess whether the magnitude of these effects differed between groups, participant-level difference scores (e.g., low-frequency minus high-frequency accuracy) were computed and compared across groups using Mann–Whitney *U*-tests. This approach allowed evaluation of group differences in sensitivity to psycholinguistic parameters while avoiding parametric interaction models that would be inappropriate given sample size and distributional constraints.

For written non-word dictation and grapheme dictation, which primarily probe sublexical phonological-to-orthographic conversion processes, between-group comparisons were performed on total accuracy scores using Mann–Whitney *U*-tests. Where applicable, exploratory within-group analyses examined the effect of syllabic length using Wilcoxon signed-rank tests.

#### 2.4.3 Qualitative error analyses

Written responses were further analyzed qualitatively using predefined error categories. Operational definitions and examples for each error type are provided in Table 3. For each participant, error proportions were calculated relative to the total number of errors produced. Given the sparse and zero-inflated distributions typical of error data, qualitative analyses were primarily descriptive. Targeted between-group comparisons were conducted using Mann–Whitney *U*-tests for theoretically motivated error categories. Effect sizes (*r*) were reported to facilitate interpretation.

**Table 3:** Qualitative error categories used for the analysis of written word production.

Error category	Operational definition	Example (French)	Corresponding example in English
Phonologically plausible errors	Incorrect spellings that preserve the phonological form of the target and conform to phoneme-to-grapheme conversion rules.	Bateau (“boat”) → bato	knife → nife
Non-phonologically plausible errors	Incorrect spellings that cannot be derived from the target's phonology and violate standard phoneme-to-grapheme correspondences.	Bateau (“boat”) → bameau	boat → boaf
Semantic errors	Production of a real word that is semantically related to the target but phonologically and orthographically unrelated.	chien (“dog”) → chat (‘cat)	dog → cat
Formal visual paragraphias	Production of a real word sharing visual–orthographic similarity with the target without semantic or phonological relatedness.	table (“table”) → cable (cable)	house → horse
Lexicalizations	Production of a real word in response to a non-word target.	plade → plage (beach)	snorp → snore
Other pooled error types	Responses not fitting the above categories, including no-response errors or visually driven errors in picture naming.	pomme (“apple”) → ∅ banane (“banana”) → lune (“moon”)	apple → ∅ banana → moon

Error categories were defined a priori and were mutually exclusive. Each written response was assigned to a single error category.

## 3 Results

Results are presented in four parts: between-group differences in cognitive screening and diagnostic measures, between-group differences in overall written word production performance, effects of psycholinguistic parameters, and qualitative analyses of error patterns.

### 3.1 Between-group differences in cognitive screening and diagnostic measures

As shown in Table 1, participants with lvPPA differed significantly from healthy controls on global cognitive screening and language measures. The lvPPA group showed significantly lower performance on the MoCA and the DTLA, reflecting impairments in overall cognitive functioning and language abilities.

Group differences were also observed on measures of verbal short-term and working memory, with participants with lvPPA performing significantly worse than controls on both digit span forward and digit span backward.

Finally, executive function measures revealed significantly longer completion times on the Alphaflex, particularly in the more demanding Part B, in the lvPPA group relative to controls. No reliable group differences were observed for error counts.

### 3.2 Between-group differences in written word production

Group comparisons on total accuracy scores revealed widespread impairments in written word production in the lvPPA group relative to healthy controls (Table 4). Participants with lvPPA performed significantly worse than controls on written picture naming, written word dictation, grapheme dictation, and the letter-in-word judgment task, with medium to large effect sizes. These group differences remained significant after correction for multiple comparisons using the Holm–Bonferroni procedure.

**Table 4:** Between-group differences in written word production (total accuracy scores).

Task (max)	HC mean (SD)	HC min–max	lvPPA mean (SD)	lvPPA min–max	*U*	*p*	*r*	*p* (Holm)
Written picture naming (24)	22.85 (1.07)	21–24	18.92 (4.17)	10–23	133.0	0.003^**^	0.598	0.008^**^
Written word dictation (47)	44.77 (1.96)	42–47	38.17 (5.24)	29–46	142.0	< 0.001^***^	0.696	0.003^**^
Written non-word dictation (15)	10.85 (1.95)	6–14	9.08 (2.39)	5–13	111.5	0.069	0.364	0.069
Grapheme dictation (30)	27.62 (1.89)	23–30	24.75 (3.25)	19–30	122.0	0.017^*^	0.479	0.034^*^
- Consonant graphemes (17)	14.85 (1.99)	10–17	13.58 (2.02)	10–17	109.0	0.093	0.34	–
- Vowel graphemes (13)	12.62 (0.51)	12–13	11.17 (1.47)	8–13	128.0	0.004^**^	0.54	–
Letter-in-word judgment (24)	23.69 (0.48)	23–24	18.92 (3.90)	12–24	137.0	< 0.001^***^	0.642	–
- Phoneme present/letter present (6)	6.00 (0.00)	6–6	4.92 (0.90)	3–6	130.0	0.007^**^	0.56	0.002^**^
- Phoneme absent/letter present (6)	5.92 (0.28)	5–6	4.67 (1.07)	2–6	138.5	< 0.001^***^	0.65	0.001^***^
- Phoneme present/letter absent (6)	5.92 (0.28)	5–6	4.33 (1.37)	1–6	122.5	0.008^**^	0.48	0.010^**^
- Phoneme absent/letter absent (6)	6.00 (0.00)	6–6	5.00 (1.48)	2–6	117.0	0.005^**^	0.42	0.010^**^

Between-group comparisons were conducted using Mann–Whitney U-tests. Effect sizes are reported as r. For the main task-level analyses, p-values were adjusted for multiple comparisons across tasks using the Holm–Bonferroni procedure. For the letter-in-word judgment subcategory analyses, Holm–Bonferroni correction was applied across the four subcategories only. Analyses comparing consonant and vowel graphemes in the grapheme dictation task were conducted as secondary, targeted analyses and were therefore not corrected for multiple comparisons. Significance levels are indicated as follows: ^*^p < 0.05, ^**^p < 0.01, ^***^p < 0.001.

In contrast, performance on written non-word dictation did not differ significantly between groups after correction, although a trend toward lower accuracy was observed in the lvPPA group, associated with a moderate effect size. Overall, this pattern indicates that written production deficits in lvPPA extend across multiple tasks engaging lexical and orthographic output processes, while sublexical spelling of non-words appears relatively less affected at the group level.

To further characterize performance on grapheme dictation, accuracy was examined separately for consonant and vowel graphemes in secondary analyses (see Table 4). Between-group comparisons revealed no significant difference between participants with lvPPA and healthy controls for consonant graphemes, although a moderate effect size was observed. In contrast, participants with lvPPA were significantly less accurate than controls for vowel graphemes, with a large effect size. Thus, the group-level impairment observed in grapheme dictation was primarily driven by reduced accuracy for vowel graphemes, whereas performance on consonant graphemes was relatively preserved.

A finer-grained analysis was conducted to examine whether group differences on the letter-in-word judgment task varied as a function of phonology–orthography correspondence (see Table 4). Accuracy was decomposed into four subcategories defined by the presence or absence of the target letter in the word's orthographic form and the presence or absence of the corresponding phoneme in the word's phonological form. Participants with lvPPA performed significantly worse than healthy controls across all four subcategories (all Holm-corrected *p* ≤ 0.010), with moderate-to-large effect sizes (*r* = 0.42–0.65). However, the largest group difference was observed in the phoneme absent/letter present condition (e.g., L in FUSIL /fyzi/), which yielded the strongest effect size. Whereas, controls performed near ceiling across all conditions, the lvPPA group showed a heterogeneous pattern of reduced accuracy across subcategories.

### 3.3 Influence of psycholinguistic parameters

Analyses examining the influence of psycholinguistic parameters revealed systematic modulation of written word production performance in the lvPPA group (Table 5). Within-group comparisons showed that participants with lvPPA were significantly less accurate for low-frequency than for high-frequency items, for orthographically irregular than for regular words, and for longer than for shorter words in both written picture naming and written word dictation. In addition, written word dictation performance in the lvPPA group was significantly influenced by imageability, with lower accuracy for words with low imageability. These effects were associated with moderate to large effect sizes. In contrast, healthy control participants showed no reliable effects of frequency, regularity, syllabic length, or imageability, consistent with near-ceiling performance across conditions.

**Table 5:** Effects of psycholinguistic parameters on written word production.

Task/Parameter	HC *p*	HC *r*	lvPPA *p*	lvPPA *r*	*U*	*p*	*p* (Holm)	*r*
Picture naming – frequency	0.705	0.649	0.009	0.838	29.5	0.007	0.058^t^	0.528
Picture naming – regularity	0.257	0.727	0.077	0.521	103.5	0.167	0.666	0.277
Picture naming – length (3 vs. 1 syll.)	0.157	0.833	0.713	0.340	78.0	1.000	1.000	0.000
Word dictation – frequency	0.004	0.824	0.001	0.849	53.5	0.187	0.561	0.267
Word dictation – regularity	0.952	0.455	0.176	0.408	50.0	0.132	0.661	0.305
Word dictation – imageability	0.002	0.882	0.001	0.838	37.0	0.024	0.144	0.446
Word dictation – length (3 vs. 1 syll.)	0.305	0.669	0.003	0.793	29.5	0.008	0.053^t^	0.528
Non-word dictation – length (3 vs. 1 syll.)	0.066	0.688	0.003	0.793	63.0	0.403	0.805	0.163

Within-group effects were tested using Wilcoxon signed-rank tests. Between-group comparisons were conducted on participant-level difference scores using Mann–Whitney U-tests. p-values in the final column were adjusted using the Holm–Bonferroni procedure. Effect sizes are reported as r. ^t^, trend toward significance.

Between-group comparisons of participant-level difference scores indicated that the magnitude of psycholinguistic effects was generally greater in the lvPPA group than in controls across tasks and parameters. Although none of these differences remained statistically significant after Holm–Bonferroni correction for multiple comparisons, the largest effects were observed for lexical frequency in picture naming and for syllabic length in written word dictation. Overall, the observed effect sizes and the consistency of effect directions across parameters indicate greater modulation of written word production by lexical, orthographic, and structural properties in lvPPA.

For written non-word dictation, exploratory analyses revealed a significant effect of syllabic length within the lvPPA group, whereas no such effect was observed in controls. Consistent with the task-level analyses, between-group comparisons of length-related difference scores did not reach statistical significance after correction.

### 3.4 Qualitative error analyses

Qualitative analyses were conducted to characterize the nature of written production errors in lvPPA and to complement the quantitative accuracy findings. Error categories and their operational definitions are provided in Table 6.

**Table 6:** Qualitative error profiles in written word production.

Task	Error type	HC mean (SD)	HC total	lvPPA mean (SD)	lvPPA total	*U*	*p*	*r*
Picture naming	Semantic	0.44 (0.40)	7	0.39 (0.33)	26	51.0	0.855	0.05
	NPPE	0.50 (0.44)	8	0.25 (0.38)	7	38.0	0.248	0.25
	PPE	0.06 (0.17)	1	0.06 (0.12)	4	60.0	0.569	0.09
	Other errors	0.00 (0.00)	0	0.30 (0.38)	24	81.0	0.018^*^	0.42
Word dictation	NPPE	0.21 (0.23)	7	0.26 (0.21)	30	61.5	0.614	0.12
	PPE	0.71 (0.30)	19	0.55 (0.27)	63	35.5	0.196	0.29
	Formal visual paragraphias	0.08 (0.13)	3	0.19 (0.28)	12	69.5	0.259	0.24
Non-word dictation	NPPE	0.92 (0.10)	49	0.93 (0.13)	64	82.0	0.818	0.04
	Lexicalizations	0.08 (0.10)	5	0.07 (0.13)	7	74.0	0.818	0.04

NPPE, non-phonologically plausible errors; PPE, phonologically plausible errors. Other errors in picture naming include no-response and visually driven errors. Values represent mean proportions (SD) of each error type relative to the total number of errors produced by each participant within a given task. Total columns report the absolute number of errors observed in each group and are provided for descriptive clinical purposes only, to reflect overall error burden. Between-group comparisons were conducted using Mann–Whitney U-tests on participant-level error proportions. Effect sizes are reported as r. ^*^p < 0.05.

Across tasks, participants with lvPPA exhibited a heterogeneous pattern of errors, with prominent contributions of phonologically plausible (e.g., Pince “pliers” → PINSE) and non-phonologically plausible (Nain “dwarf” → NOIN) spelling errors. Phonologically plausible errors were particularly frequent in written word dictation and, to a lesser extent, in written picture naming, consistent with relatively preserved access to phonological representations in the context of impaired orthographic retrieval. Non-phonologically plausible errors were also observed across tasks and were especially prevalent in non-word dictation, reflecting disruptions in sublexical phonological-to-orthographic conversion and in the serial maintenance of graphemic representations.

Semantic errors (e.g., tigre “tiger” → ZEBRE “zebra”) and formal visual paragraphias (e.g., bribe “snippet” → BRIDE “bridle”) were less frequent overall but were mainly confined to lexical tasks, namely written picture naming and written word dictation. This pattern is consistent with impairments affecting lexical–semantic access and orthographic representations during written word production. In contrast, lexicalizations (e.g., fal → SALE “dirty”) were observed almost exclusively in written non-word dictation, indicating intrusion of lexical representations during tasks that primarily rely on sublexical spelling mechanisms.

Healthy control participants produced very few errors across tasks, resulting in limited variability in error proportions. When errors occurred, they were predominantly classified within pooled categories (e.g., no-response or visually driven errors) and did not show a consistent distribution across specific error types. Consequently, between-group comparisons of error proportions were restricted to theoretically motivated categories and were interpreted descriptively, with effect sizes reported to facilitate interpretation alongside the overall distribution of errors.

## 4 Discussion

The present study provides a detailed characterization of written word production deficits in lvPPA by combining global task-level analyses with finer-grained examinations of orthographic and phonology–orthography correspondence processes. Relative to healthy controls, participants with lvPPA showed marked impairments across multiple written production tasks, including written picture naming, written word dictation, grapheme dictation, and letter-in-word judgment, whereas performance on written non-word dictation was comparatively preserved at the group level.

Beyond these global differences, secondary analyses revealed a non-uniform pattern of impairment. In grapheme dictation, group differences were primarily driven by reduced accuracy for vowel graphemes, with consonant graphemes remaining relatively preserved. In the letter-in-word judgment task, the largest group difference was observed in the condition where the target letter was present in the orthographic form but absent from the phonological form, highlighting a particular vulnerability when orthographic knowledge must be accessed independently of phonological support. These findings indicate that written production deficits in lvPPA cannot be reduced to a single locus of impairment but instead reflect selective vulnerabilities within orthographic processing and phonology–orthography mapping mechanisms. Importantly, these written language impairments should be interpreted within the broader language profile characteristic of lvPPA. This variant is primarily associated with degeneration of left temporoparietal language networks, which support phonological processing and verbal working memory (Whitwell et al., 2015; Montembeault et al., 2018), two functions that are essential for the coordination of phonological, lexical, and orthographic representations during written word production.

A useful framework for interpreting this pattern is provided by cognitive classifications of acquired agraphia (Denes et al., 2020), particularly the distinction between phonological, surface, and deep agraphia. The constellation of findings observed here is most consistent with a subset of features typically associated with deep agraphia, rather than with a purely phonological or surface profile. Crucially, deep agraphia has been characterized by impaired access to orthographic representations when phonological support is reduced or unreliable, often accompanied by phonologically implausible errors and difficulty retrieving orthographic knowledge independently of phonology (Bub and Kertesz, 1982).

In the present study, the relative preservation of written non-word dictation at the group level argues against a primary phonological agraphia (Shallice, 1981), while the selective vulnerability of vowel graphemes and the pronounced impairment in the phoneme-absent/letter-present condition suggest reduced availability or stability of abstract orthographic representations (Rapp and Caramazza, 1997; Kemény and Landerl, 2021). Crucially, this condition requires explicit access to orthographic knowledge when phonological cues are non-informative, a demand that overlaps with aspects of deep agraphia as described in the neuropsychological literature (Bub and Kertesz, 1982; Tiu et al., 2025). Although tasks such as grapheme dictation and letter-in-word judgment may place demands on short-term or working memory processes, the selective nature of the impairments observed here suggests that memory limitations alone cannot account for the pattern of results, instead pointing to specific vulnerabilities in orthographic processing. Thus, the lvPPA profile observed here is best described as deep-agraphia–like, reflecting a disruption in the interaction between orthographic representations and phonological processes rather than a categorical agraphic syndrome. Importantly, the present use of the term deep-agraphia–like is intended as a functional descriptor rather than a categorical diagnosis, referring to shared computational constraints on orthographic access rather than to the full clinical syndrome as classically described in focal lesion studies.

The selective vulnerability of vowel graphemes further refines this orthographic interpretation. In French, vowel phonemes are characterized by a high degree of phoneme–grapheme ambiguity, resulting in substantial inconsistency in the phonology-to-orthography direction that disproportionately affects vowels (Ziegler et al., 1996; Peereman and Content, 1999). Consequently, accurate vowel spelling places heavier demands on access to abstract orthographic representations beyond what can be achieved through sublexical phonological conversion alone (Rapp and Caramazza, 1997; Tao and Rapp, 2025). By contrast, consonant phonemes typically involve more constrained phoneme–grapheme correspondences and thus lower demands on orthographic selection.

Although direct neuroanatomical correlates of orthographic consonant and vowel representations remain poorly specified, neuropsychological evidence from acquired dysgraphia indicates that these two classes of graphemes can be selectively impaired, implying partially distinct representational substrates within the orthographic system (Cubelli, 1991; Miceli et al., 2004; Buchwald and Rapp, 2006). In the context of lvPPA, which preferentially affects temporoparietal language networks (Mandelli et al., 2023) known to support abstract orthographic representations (Rapp et al., 2016; Tao and Rapp, 2025), such an organization may render vowel representations, characterized by greater orthographic ambiguity, particularly vulnerable. This interpretation does not imply a strict anatomical segregation of vowel and consonant representations, but rather differential vulnerability of representational properties that rely more heavily on abstract orthographic specification.

Within this framework, the selective vulnerability observed for vowel graphemes and the marked impairment in the phoneme-absent/letter-present condition of the letter-in-word judgment task likely reflect a common underlying constraint: increased reliance on abstract orthographic representations when phonological information is insufficient or unreliable. Consistent with this interpretation, the largest group difference was observed precisely in this condition, which requires explicit access to orthographic knowledge when phonological cues are non-informative (Kemény and Landerl, 2021). Together, these findings suggest that vowel processing may constitute a particularly sensitive marker of orthographic vulnerability in lvPPA, reflecting reduced availability or stability of orthographic representations rather than a primary phonological deficit.

A growing body of work has documented spelling and writing impairments in the lvPPA, although findings remain heterogeneous with respect to the underlying cognitive mechanisms. Across group and case-series studies, individuals with lvPPA have been shown to exhibit mixed patterns of agraphia, variably affecting lexical spelling, phoneme–grapheme conversion, and orthographic working memory, rather than a uniform impairment of a single spelling route (Sepelyak et al., 2011; Shim et al., 2012; Faria et al., 2013). Several studies have reported comparable difficulty for real words and non-words, with both phonologically plausible and implausible errors, suggesting concurrent disruption of phonological and orthographic processes (Sepelyak et al., 2011; Neophytou et al., 2019). Others have emphasized the sensitivity of spelling performance to psycholinguistic variables such as regularity, age of acquisition, orthographic neighborhood density, and semantic properties, underscoring the contribution of lexical–orthographic knowledge in lvPPA (Carroll-Duhigg et al., 2025; Nickels and Kielar, 2025).

Importantly, spelling has also been shown to provide diagnostically relevant information in PPA, with lvPPA displaying profiles that differ from both semantic and non-fluent variants but that do not map cleanly onto classical categories of acquired agraphia (Neophytou et al., 2019). The present study extends this literature by moving beyond global accuracy and lexicality effects to identify specific conditions that are particularly sensitive to orthographic vulnerability in lvPPA. By demonstrating selective impairment for vowel graphemes and a pronounced deficit in the phoneme-absent/letter-present condition of a letter-in-word judgment task, our findings provide direct evidence for reduced availability or stability of abstract orthographic representations when phonological cues are non-informative. This fine-grained characterization refines previous descriptions of agraphia in lvPPA and helps reconcile heterogeneous findings by highlighting the role of orthographic ambiguity and orthography–phonology interaction as key determinants of written production deficits in this variant.

The present findings underscore the importance of moving beyond global accuracy measures when assessing written language in lvPPA. Previous work has shown that spelling impairments in this variant are heterogeneous and do not consistently align with a single agraphic profile, limiting the clinical interpretability of standard dictation tasks alone (Sepelyak et al., 2011; Shim et al., 2012; Neophytou et al., 2019). The current results indicate that fine-grained task manipulations are needed to reveal specific vulnerabilities within the writing system.

In particular, the marked impairment observed in conditions requiring access to orthographic knowledge in the absence of informative phonological cues highlights the clinical value of tasks that minimize phoneme–grapheme conversion demands. The letter-in-word judgment task illustrates how orthographic knowledge can be probed without requiring full written output, thereby reducing confounds related to motor execution or graphemic buffer limitations. In addition, the selective vulnerability of vowel graphemes suggests that vowel-based measures may provide a sensitive marker of orthographic fragility in lvPPA, consistent with evidence that spelling performance in PPA is modulated by orthographic complexity and psycholinguistic variables (Macoir et al., 2021; Carroll-Duhigg et al., 2025).

Together, these findings support a clinical assessment approach that complements traditional dictation tasks with targeted measures probing orthographic processing under reduced phonological support, with potential benefits for diagnostic sensitivity and longitudinal monitoring in lvPPA.

### 4.1 Limitations and future directions

Several limitations should be acknowledged. First, the relatively modest sample size and the heterogeneity inherent to lvPPA limit the generalizability of the findings and may have reduced sensitivity to more subtle subgroup effects. Second, the cross-sectional design precludes conclusions regarding the evolution of written production deficits over time and their relationship to disease progression. Longitudinal studies will be necessary to determine whether the orthographic vulnerabilities identified here, particularly those affecting vowel graphemes and phonology-independent orthographic access, emerge early in the disease course or reflect later-stage changes. In addition, although time since diagnosis was documented for all participants, the cross-sectional design and modest sample size prevented reliable examination of the relationship between disease duration and written language performance. Longitudinal studies will therefore be necessary to clarify how orthographic impairments evolve across different stages of lvPPA.

In addition, the absence of direct neuroanatomical correlates prevents firm conclusions about the neural substrates underlying the observed written language impairments. Future work combining fine-grained behavioral measures of written production with structural and functional neuroimaging could help clarify the contribution of temporoparietal and ventral language networks to orthographic processing deficits in lvPPA. Finally, extending this approach to other PPA variants and to additional writing tasks, including connected writing and ecological measures, would further inform the specificity and clinical utility of the proposed assessment framework.

## 5 Conclusion

To conclude, this study demonstrates that written production deficits in lvPPA extend beyond global accuracy reductions and reflect selective vulnerabilities within orthographic processing, particularly under conditions of reduced phonological support. By identifying specific orthographic constraints, most notably involving vowel graphemes and phonology-independent access to orthographic knowledge, these findings refine current characterizations of agraphia in lvPPA and underscore the clinical value of fine-grained assessment of written language.

## Data Availability

The raw data supporting the conclusions of this article will be made available by the authors, without undue reservation.
